# Cerebral Infarction as an Initial Manifestation in Acute Promyelocytic Leukemia and Deterioration After All-Trans Retinoic Acid Treatment

**DOI:** 10.7759/cureus.70563

**Published:** 2024-09-30

**Authors:** Daisuke Suzuki, Kenji Kikuchi, Kunie Sugasawa, Soichi Saito, Yoshihiro Suzuki

**Affiliations:** 1 Neurology, Nihonkai General Hospital, Sakata, JPN; 2 Hematology, Nihonkai General Hospital, Sakata, JPN

**Keywords:** acute cerebral infarction, acute promyelocytic leukemia (apl), all-trans retinoic acid (atra), disseminated intravascular coagulation (dic), thrombosis

## Abstract

There are neither predictive tests nor preventive strategies/treatments for acute promyelocytic leukemia (APL)-associated bleeding/thrombosis incidence. We encountered the case of a woman in her 70s who was admitted due to sudden-onset right hemiparesis. The patient was diagnosed with acute cerebral infarction as the initial manifestation of APL. Intravenous recombinant human soluble thrombomodulin was initiated on admission, followed by oral all-trans retinoic acid two days later. However, the patient’s condition deteriorated due to APL-associated diphasic cerebral infarction with left internal carotid artery occlusion, and she died 10 days after admission. Thus, the degree of main artery stenosis should be evaluated before treatment in patients with APL who have coagulopathy.

## Introduction

Cerebrovascular thrombotic events associated with cancer, known as Trousseau syndrome, are often caused by coagulopathy related to malignancies. This coagulopathy can occur either before diagnosis or after cancer has been established [1,2]. Hematologic malignancies may also lead to coagulopathy; however, bleeding is more common than thrombosis as a complication, especially in acute myeloid leukemia (AML) [3].

Acute promyelocytic leukemia (APL) is a subset of AML characterized by abnormal hypergranular promyelocytes in the bone marrow and peripheral blood and consumptive coagulopathy [4]. APL is relatively rare and comprises 7%-8% of cases of adult AML [4]. APL-related coagulopathy, including bleeding/thrombosis, is a major problem, sometimes acquiring a life-threatening status [5]. In APL, bleeding complications are relatively more common than thromboses; however, they appear to be more frequent than previously thought [5]. APL-related thrombosis can occur in various organs with usual symptoms including deep venous thrombosis, pulmonary embolism, myocardial infarction, and cerebral stroke [5]. APL is treated with all-trans retinoic acid (ATRA), arsenic trioxide, or chemotherapy with or without supportive treatment [4]. APL is a very aggressive malignancy with a median survival of less than one month without treatment, and the prognosis of APL has markedly improved with these treatment options [4]. However, despite ATRA, early deaths due to APL-related coagulopathy (bleeding/thrombosis) still occur [6]. Nevertheless, the management of APL-related thrombosis also remains challenging due to the lack of available information and unknown complex pathogenetic mechanisms [5,7].

Several studies have evaluated risk factors for early death in APL with thrombotic events; however, large-scale studies have not been conducted due to the small number of cases [6,8,9]. Herein, we report a rare case of APL-associated diphasic cerebral infarction, with the first attack as the initial manifestation of APL and the second as a side effect of ATRA. We detected severe stenosis of the internal carotid artery before the occlusion, which may lead to future studies elucidating the pathophysiology of APL-related thrombosis.

## Case presentation

A woman in her 70s with a history of hypertension, hyperlipidemia, and type 2 diabetes was admitted with sudden-onset right hemiparesis. Neurological examination revealed moderate right hemiparesis and mild word-finding difficulties. Laboratory examination revealed severe leukopenia and mild thrombocytopenia, with myeloblasts present. The abnormal hemocoagulation panels showed a slightly prolonged prothrombin time-international normalized ratio, low fibrinogen levels, and elevated D-dimer and fibrin degradation product levels, indicating disseminated intravascular coagulation (DIC) (Table 1).

**Table 1:** Complete blood count and coagulation panels. Segment, segmental neutrophils; Lymph, lymphocytes; Mono, monocytes; Eos, eosinophils; Baso, basophils; Hb, hemoglobin; Hct, hematocrit; Plt, platelet; PT-INR, prothrombin time-international normalized ratio; PTT, partial thromboplastin time; FDP, fibrin degradation products; ATRA, all-trans retinoic acid

Laboratory items	Laboratory value (day 1; on admission)	Laboratory value (day 4; after initiating ATRA treatment)	Laboratory value (day 7; on deterioration)	Normal range
Complete blood count				
White blood cells (×10^3^/µL)	0.98	1.84	1.77	3.30-8.60
Segment (%)	51	48	54	30-65
Lymph (%)	25	20	29	20-50
Mono (%)	4	1	4	2-10
Eos (%)	1	0	3	0-5
Baso (%)	3	0	8	0-2
Blasts (%)	16	31	2	0
Red blood cells (×10^6^/µL)	4.47	3.53	3.51	3.86-4.92
Hb (g/dL)	13.2	10.4	10.3	11.6-14.8
Hct (%)	38.4	29.7	30.1	35.1-44.4
Plt (×10^3^/µL)	120	94	111	158-348
Coagulation panel				
PT-INR	1.26	1.26	1.11	0.90-1.10
PTT (seconds)	21.7	24.8	26.3	23.0-34.9
Fibrinogen (mg/dL)	144	244	425	188-355
D-dimer (µg/mL)	15.50	11.60	5.09	<1.00
FDP (µg/mL)	63.5	44.7	12.8	<5.0

**Figure 1:**
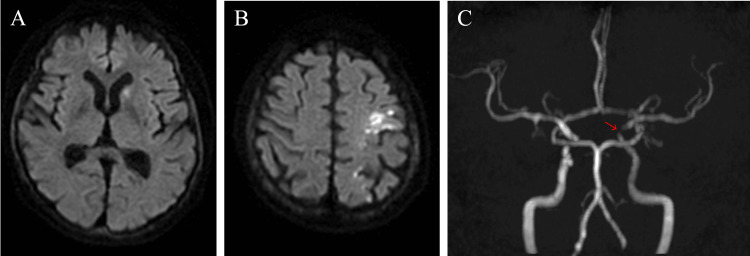
(A and B) Brain magnetic resonance imaging and (C) angiography at admission. Hyperintense lesions in the territory of the anterior branch of the left middle cerebral artery on axial diffusion-weighted imaging (A and B) and severe stenosis of the left internal carotid artery (C, arrow).

Brain diffusion-weighted magnetic resonance imaging revealed hyperintensity in the left middle cerebral artery territory (Figures 1A-1B) and magnetic resonance angiography revealed left internal carotid artery stenosis (Figure 1C, arrow).

**Figure 2:**
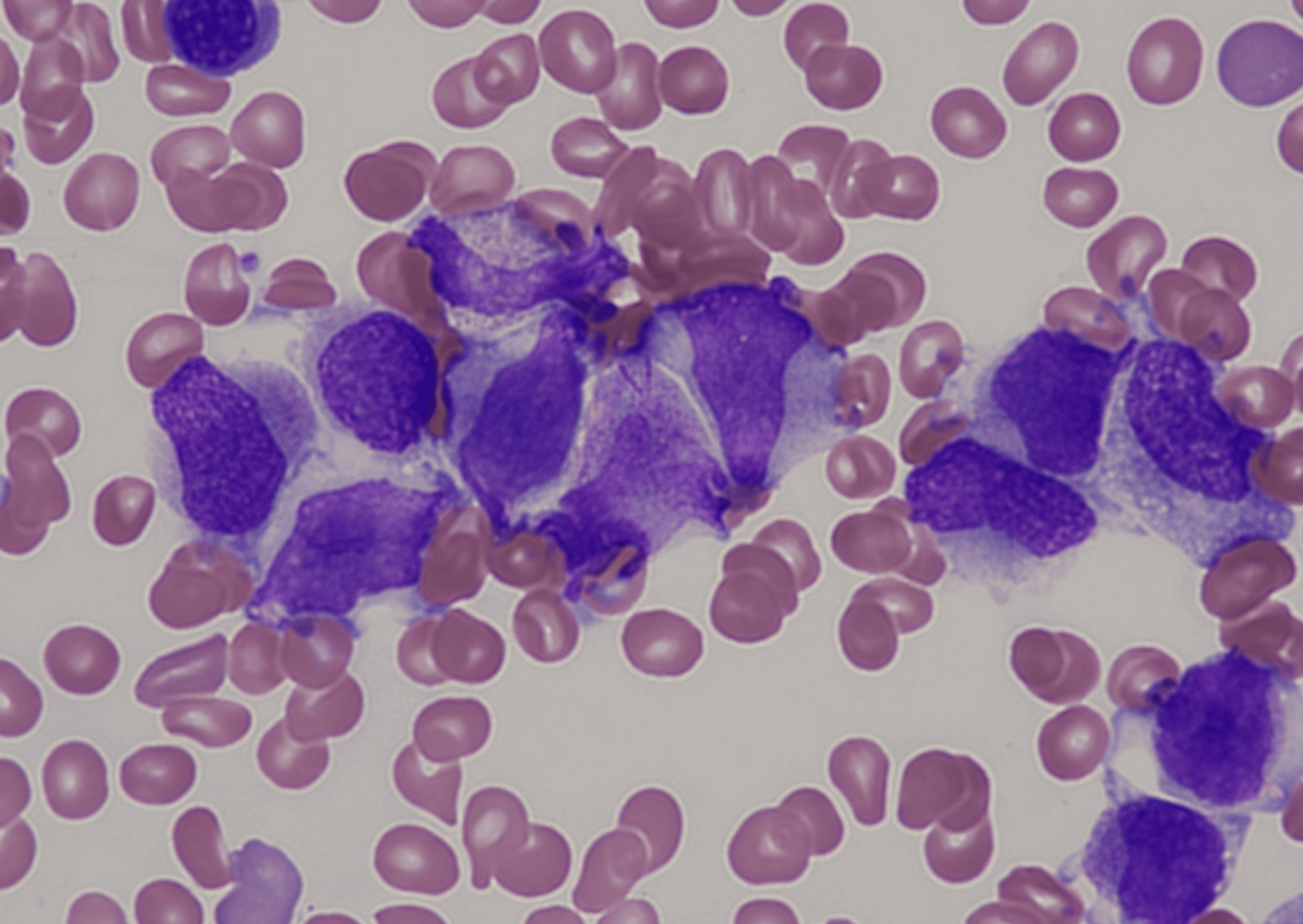
Bone marrow aspiration. Infiltration of abnormal promyelocytes with or without Auer rods.

Bone marrow aspiration revealed infiltration of abnormal promyelocytes with or without Auer rods (Figure 2).

**Figure 3:**
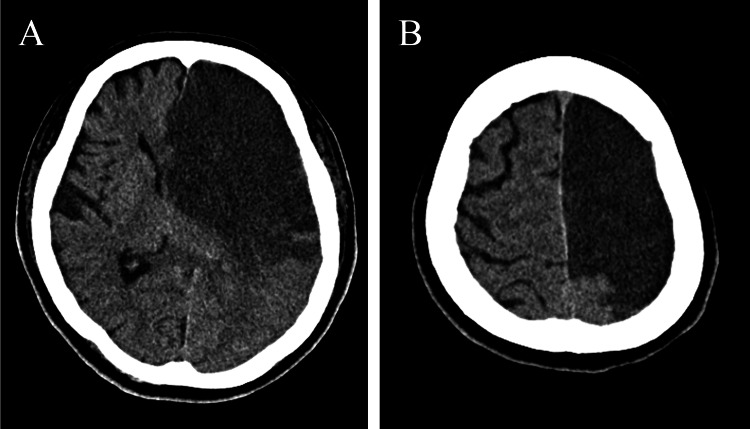
(A and B) Brain computed tomography on deterioration in condition after treatment. Expanding acute cerebral infarction lesion in the left internal carotid artery (A and B).

Molecular examination revealed promyelocytic leukemia (PML)-retinoic acid receptor alpha (RARα) fusion protein by reverse-transcription polymerase chain reaction (RT-PCR). The patient was diagnosed with acute cerebral infarction as the initial manifestation of APL. Intravenous recombinant human soluble thrombomodulin (daily dose of 19,200 U) was initiated upon admission due to concerns about bleeding caused by thrombocytopenia in APL. This was followed by oral ATRA (daily dose of 60 mg) three days after admission. However, four days after initiating ATRA (seven days after admission), the right hemiparesis and level of consciousness worsened. A brain computed tomography scan revealed an expansion of the infarction area in the left internal carotid artery with cerebral herniation (Figures 3A-3B), while a follow-up laboratory examination showed a trend toward improvement in complete blood counts and hemocoagulation panels. The patient’s condition deteriorated, and she died 10 days after admission.

## Discussion

The present case was a unique occurrence of APL-associated diphasic cerebral infarction. Severe stenosis of the left internal carotid artery was detected at the first attack as an initial manifestation of APL before treatment; however, it became completely occluded after ATRA treatment. This is an instructive case, possibly indicating the necessity to evaluate the main artery before treatment in patients with APL and thrombotic tendencies.

APL is a distinguished subtype of AML characterized by a specific genetic alteration of t(15;17)(q22;q21), PML-RARα fusion, and proliferation of promyelocytes in peripheral blood and bone marrow [4]. APL is distinguished from other myelocytic leukemias because of its high cure rate with treatment [4]. ATRA, known as one of the differentiation therapies, is the mainstay in the treatment of APL [4]; however, treatment with ATRA alone results in a poor prognosis. Therefore, ATRA is used in combination with anthracycline-based regimens as consolidation therapy in APL, leading to a complete response in 90% of cases [10]. Supportive therapy also plays an important role in the treatment of APL. This includes managing bleeding complications due to DIC and systemic infections resulting from an immunosuppressed status [4]. In the former case, platelet counts should be maintained above 30-50 × 10^3^/µL through platelet transfusions, and fibrinogen levels should also be maintained above 150 mg/dL with fresh frozen plasma [4]. There is no consensus on whether DIC associated with APL should be treated with heparin, thrombomodulin, or anti-fibrinolytic; however, different therapeutic regimens should consider their impact on the coagulation-anticoagulation balance in patients with APL [11]. In the latter case, APL patients with fever should receive an empiric antibiotic and antifungal regimen based on appropriate laboratory tests [4].

APL usually presents with laboratory findings of DIC because of the complex interaction between abnormal promyelocytes and the coagulation system, leading to life-threatening APL-related coagulopathy [12]. Bleeding complications, such as intracranial hemorrhages, gastrointestinal bleeding, and pulmonary intra-alveolar hemorrhage, are common in APL due to thrombocytopenia and increased procoagulant activities derived from leukemic promyelocytes [3]. However, thrombotic complications, such as deep venous thrombosis, pulmonary embolism, myocardial infarction, and cerebral stroke, paradoxically occur in APL and are less common than APL-related bleeding [3].

APL-associated thrombosis could occur pre-induction (sometimes as an initial manifestation in APL), during induction, or post-induction [12]; however, thrombotic complications are more likely to occur in earlier stages [8]. Long-term outcomes improve with ATRA treatment, but the early mortality rate associated with APL-related thrombosis remains high over time despite ATRA treatment [4,13]. There are no preventive strategies, predictive factors, or treatments for APL-related coagulopathy due to its unknown complex etiology [12].

There are several plausible explanations for the thrombotic tendency in APL, including APL itself, therapeutic agents of APL (ATRA, chemotherapy, or antifibrinolytic drugs), and ATRA syndrome [12]. First, leukemic promyelocytes release potent procoagulant factors, including tissue factor (TF), a membrane protein, leading to the activation of the coagulation cascade [5,14]. Whether or not either bleeding or thrombotic complications happen depends on the balance between profibrinolytic and antifibrinolytic factors in APL [3]. Second, increased apoptosis of promyelocytes due to the induction of treatment might also lead to increased activation of TF [3]. ATRA itself also increases endothelial TF expression mediated by interleukin-1β in vitro, whereas ATRA results in a rapid downregulation of TF and normal coagulative status within several days, possibly followed by coagulation [15]. Third, ATRA syndrome is characterized by the infiltration of maturing myeloid cells in various organs, which results in thromboses due to leukocytosis [3].

On the other hand, TF, expressed in normal tissue and malignant cells, can be induced by interleukin-1β or low-density lipoprotein cholesterol in intravascular plaques [16,17]. High levels of TF antigen and activation are seen in local atherosclerotic lesions, which cause further progression of these lesions [17].

TF, APL, and ATRA may also impact severe arteriosclerosis, with possible internal carotid artery occlusion after ATRA treatment. However, there are no studies evaluating the main artery in patients with APL before severe thrombosis complications. The present report comprises a single case, and large-scale studies are essential to validate our findings.

## Conclusions

Neurologists should be aware of possible cerebral infarction in patients with APL, while hematologists should be informed about predicting coagulative complications by evaluating main artery stenosis before treatment. Further studies are needed to elucidate the pathophysiology of APL-related thrombosis.
